# Rapid intra-adrenal feedback regulation of glucocorticoid synthesis

**DOI:** 10.1098/rsif.2014.0875

**Published:** 2015-01-06

**Authors:** J. J. Walker, F. Spiga, R. Gupta, Z. Zhao, S. L. Lightman, J. R. Terry

**Affiliations:** 1 College of Engineering, Mathematics and Physical Sciences, University of Exeter, Exeter EX4 4QF, UK; 2 Henry Wellcome Laboratories for Integrative Neuroscience and Endocrinology, University of Bristol, Whitson St., Bristol BS1 3NY, UK

**Keywords:** hypothalamic–pituitary–adrenal axis, mathematical modelling, adrenal gland, stress response, glucocorticoids

## Abstract

The hypothalamic–pituitary–adrenal axis is a vital neuroendocrine system that regulates the secretion of glucocorticoid hormones from the adrenal glands. This system is characterized by a dynamic ultradian hormonal oscillation, and in addition is highly responsive to stressful stimuli. We have recently shown that a primary mechanism generating this ultradian rhythm is a systems-level interaction where adrenocorticotrophin hormone (ACTH) released from the pituitary stimulates the secretion of adrenal glucocorticoids, which in turn feedback at the level of the pituitary to rapidly inhibit ACTH secretion. In this study, we combine experimental physiology and mathematical modelling to investigate intra-adrenal mechanisms regulating glucocorticoid synthesis. Our modelling results suggest that glucocorticoids can inhibit their own synthesis through a very rapid (within minutes), presumably non-genomic, intra-adrenal pathway. We present further evidence for the existence of a short time delay in this intra-adrenal inhibition, and also that at the initiation of each ACTH stimulus, this local feedback mechanism is rapidly antagonized, presumably via activation of the specific ACTH receptor (MC2R) signalling pathway. This mechanism of intra-adrenal inhibition enables the gland to rapidly release glucocorticoids while at the same time preventing uncontrolled release of glucocorticoids in response to large surges in ACTH associated with stress.

## 1. Introduction

**Figure 1.**
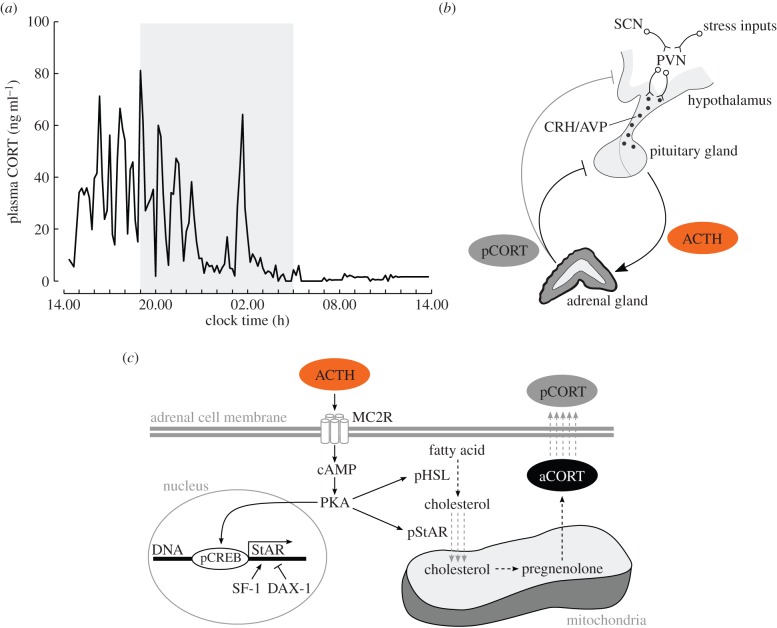
(*a*) Under basal (i.e. unstressed) conditions, glucocorticoid levels in plasma (pCORT) are characterized by both a circadian and an approximately hourly ultradian rhythm. Shaded region indicates the dark phase. Data adapted from Walker *et al.* [2]. (*b*) Regulation of HPA axis activity. The hypothalamic PVN receives circadian input from the SCN as well as stress inputs from the brainstem and from regions of the limbic system such as the hippocampus and amygdala. The PVN projects to the median eminence where it releases CRH and AVP into the hypothalamic–pituitary portal circulation. CRH and AVP pass through this vascular route to access corticotroph cells in the anterior pituitary, which respond with the rapid release of ACTH from preformed vesicles into the general blood circulation. In turn, ACTH reaches the adrenal gland where it activates the synthesis of glucocorticoid hormones. Once synthesized, glucocorticoids are rapidly released into the general circulation (pCORT) via which they reach target tissues. Glucocorticoids regulate the activity of the HPA axis, and thus their own production, through feedback mechanisms acting at the level of the pituitary gland where they inhibit ACTH release, and at the level of the PVN where they inhibit the release of CRH and AVP. (*c*) Schematic of the complex adrenal steroidogenic network. ACTH increases adrenal gland activity via PKA activation leading to non-genomic regulation of steroidogenic proteins. This includes phosphorylation of hormone sensitive lipase (HSL), a protein that increases the levels of intracellular cholesterol (the precursor of steroid hormones), and phosphorylation of steroidogenic acute regulatory protein (StAR), which promotes the transport of cholesterol into the mitochondria, where cholesterol is converted into pregnenolone by the enzyme side-chain cleavage cytochrome P450 (P450scc). This process is followed by a number of enzymatic reactions within the mitochondria and the endoplasmic reticulum that ultimately leads to glucocorticoid synthesis within the cell (aCORT), which, in turn, is released into the general blood circulation (pCORT). PKA also mediates adrenal genomic activity by inducing StAR transcription, which is, in turn, enhanced or repressed by the transcriptional regulators steroidogenic factor 1 (SF-1) and DAX-1, respectively.

The hypothalamic–pituitary–adrenal (HPA) axis is critical for the maintenance of homeostasis, regulating the hormonal response to both acute and chronic stressors. This neuroendocrine system governs these responses through the secretion of glucocorticoid hormones (cortisol in man and corticosterone in rodents; herein referred to as CORT) that are released from the adrenal glands in a highly dynamic manner, displaying both circadian and ultradian (near hourly) rhythms in the rat [1] (figure 1*a*). It is well known that the circadian profile of CORT is regulated by inputs from the suprachiasmatic nucleus (SCN) to the paraventricular nucleus (PVN) of the hypothalamus [3], where parvocellular neurons project to the median eminence of the hypothalamus and release corticotrophin-releasing hormone (CRH) and arginine vasopressin (AVP). These hormones travel through the portal veins to reach the anterior pituitary where they activate corticotroph cells to secrete adrenocorticotrophin hormone (ACTH) into the general circulation. Within cells of the adrenal cortex, ACTH activates a rapid signalling pathway that regulates the synthesis and release of CORT (figure 1*b*). Once released from the adrenal glands into the blood circulation, CORT accesses target tissues, such as the liver, and the heart and vascular tissues to exert metabolic and cardiovascular effects, respectively. Additionally, CORT modulates multiple brain structures to promote, for example, cognitive processes necessary to cope with a threatening situation (see [4] for a comprehensive review).

Owing to its lipophilic nature, CORT cannot be pre-synthesized and stored in adrenal cells, but has to be rapidly (i.e. within minutes) synthesized upon ACTH stimulation (figure 1*c*). Following the binding of ACTH to its specific melanocortin 2 receptor (MC2R) in the *zona fasciculata* of the adrenal cortex, there is increased protein kinase A (PKA)-mediated phosphorylation of steroidogenic proteins, including the rate-limiting protein StAR [5], which promotes the transport of cholesterol, the precursor of steroid hormones, inside the mitochondria [6], where a number of enzymatic reactions leads to glucocorticoid synthesis. In addition to its rapid non-genomic effects, ACTH also regulates adrenal activity by inducing the transcription of steroidogenic genes, including StAR and MC2R (figure 1*c*). Consistent with the ultradian rhythm of CORT, transcription of StAR and other steroidogenic genes also appears to be pulsatile [7]. This sequence of feed-forward mechanisms within the adrenal cortex, and more generally within the whole HPA system, is balanced by negative feedback of CORT acting at both the anterior pituitary and within the brain to inhibit further release of ACTH and CRH [8,9] (figure 1*b*).

The feed-forward–feedback interplay between the anterior pituitary and adrenal glands has been shown to be critical for the rapid ultradian ACTH and CORT oscillations observed in the blood. In contrast to the classically held notion of a hypothalamic pulse generator, we have recently shown theoretically that this feed-forward–feedback interplay can generate ultradian oscillations of CORT secretion in the presence of constant levels of CRH [10]. This mathematical hypothesis has been supported by a series of *in vivo* experiments where CRH was infused at constant levels during the circadian nadir of HPA activity and hourly pulses in both ACTH and CORT were observed [2]. Significantly, these ultradian rhythms have been shown to be important in determining the stress responsiveness of the HPA axis as a whole. For example, the behavioural response to stress has been observed to desensitize when the hourly rhythm is replaced by an equivalent constant level of CORT [11]. Furthermore, the timing of an incoming stressor, relative to the phase of an endogenous pulse of CORT, has been shown to govern the amplitude of the subsequent stress response as well as the timing of subsequent pulses [1,12].

While the effect of CORT feedback at the level of the pituitary and brain has received much attention, little is known about intra-adrenal mechanisms through which CORT may autoregulate its own synthesis and secretion. There is evidence that the glucocorticoid receptor (GR) is expressed in the adrenal cortex of both the rat [13] and man [14], and that its functionality is similar to that observed in other tissues [15]. Furthermore, a number of *in vitro* and *in vivo* studies have shown that prior stimulation of the adrenal gland results in a decreased response to further stimuli. For example, adding high concentrations of CORT to the medium of cultured adrenal cells has been shown to inhibit ACTH-stimulated CORT synthesis [16], and this effect can be seen within 1–2 h of CORT exposure [17]. These findings are consistent with studies showing that adrenals collected from hypophysectomized rats treated with CORT have lower responses to ACTH when compared with those from untreated rats [18]. Similarly, a rapid inhibition of ACTH-induced adrenal steroidogenesis has been observed following repeated adrenal stimulation with ether and ACTH [19]. Other studies have shown that there is no increase in CORT concentration in adrenal vein effluent in response to ACTH following pre-treatment with CORT [20,21]. These findings are consistent with studies showing that in rats previously exposed to a stressor, or injected with a high concentration of ACTH, adrenal CORT is not increased in response to further stimuli, suggesting that CORT synthesis is dependent on the prior state within the adrenal of the rat [22,23]. Collectively, these studies provide support for the concept that rising CORT levels within the adrenal might regulate further glucocorticoid synthesis and secretion through local activation of GR.

To investigate further the role of intra-adrenal CORT autoregulation, in this study we pursue a systems biology approach; integrating a mathematical model with *in vivo* experimental data to investigate whether rapid intra-adrenal inhibition is an important factor regulating glucocorticoid synthesis over the timescales of both the basal ultradian rhythmicity of the HPA axis and the glucocorticoid stress response. To do so, plasma ACTH, plasma CORT, and adrenal CORT were measured in rats in which rapid secretion of CORT was induced by either constant CRH infusion or intravenous (i.v.) administration of ACTH (to stimulate an ultradian CORT pulse), or in rats exposed to a mild stress. Plasma ACTH and CORT data were then used as inputs into mathematical models of adrenal CORT synthesis. Our analysis provides evidence for the existence of intra-adrenal inhibition of CORT synthesis, and further that this intra-adrenal feedback is rapidly antagonized by ACTH, presumably via activation of MC2R, effectively disinhibiting the system and enabling a rapid early response of CORT to ACTH, which remains closely regulated by subsequent steroidogenic activity within the adrenal gland.

## 2. Material and methods

### 2.1. Experimental procedures

**Figure 2.**
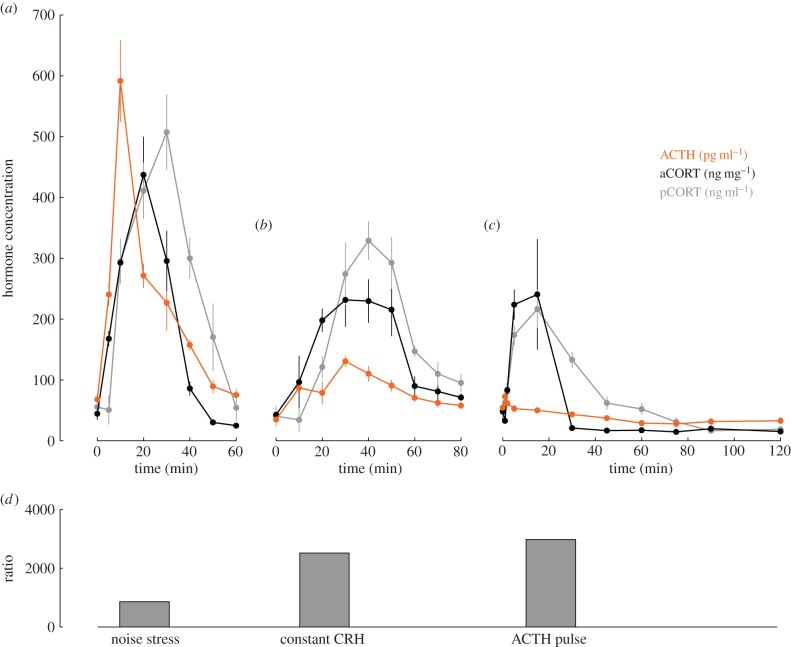
Dynamics of ACTH (orange), adrenal CORT (black) and plasma CORT (grey) during exposure to (*a*) a mild stress (noise stress), (*b*) intravenous constant CRH infusion (constant CRH) and (*c*) intravenous ACTH injection (ACTH pulse). Each data point represents the mean ± s.e.m. from *n* = 4–9 rats. For each experiment, the ratio of peak plasma CORT to peak ACTH is shown in (*d*).

Male adult Sprague Dawley rats were implanted with a double indwelling cannula in the jugular vein, as previously described [2]. Five to seven days after surgery, rats were either exposed to an acute noise stress (white noise, 110 dB, 10 min) [24,25], infused with constant CRH (0.5 µg h^−1^) [2] or injected with ACTH (10 ng per 0.1 ml, i.v.) [26]. Trunk blood and adrenal glands were collected prior to, during and after each treatment at specific time points. Plasma and adrenal hormone levels were measured using radioimmunoassay as previously described [26]. Figure 2 illustrates the hormone dynamics obtained from each of these paradigms.

### 2.2. Mathematical models and analysis

**Figure 3.**
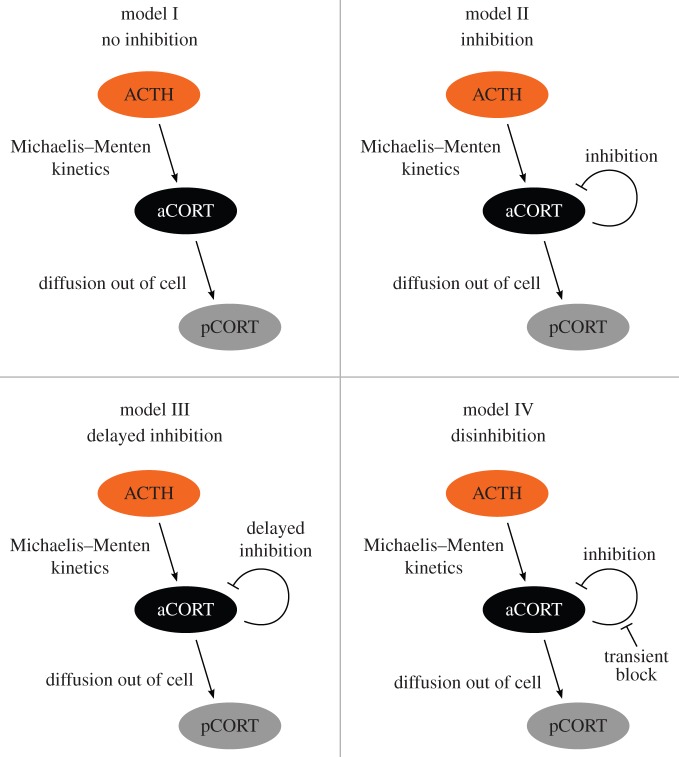
Four candidate models for adrenal CORT autoregulation. In model I (no inhibition), adrenal CORT does not regulate its own production; in model II, (inhibition), adrenal CORT regulates its own production via self-inhibition; in model III (delayed inhibition), adrenal CORT regulates its own production via delayed self-inhibition; in model IV (disinhibition), adrenal CORT regulates its own production via self-inhibition that, in turn, is initially blocked. In each panel, aCORT represents adrenal CORT, and pCORT represents plasma CORT.

We used ordinary differential equations (ODEs) to describe three candidate systems-level mechanisms for CORT autoregulation within the adrenal network as well as the null hypothesis of no CORT-driven mechanism (illustrated schematically in figure 3). The three mechanisms were either instantaneous or delayed CORT inhibition, as well as the possibility that a CORT-driven inhibitory mechanism is itself transiently blocked (that we term disinhibition). These equations take into account the synthesis and secretion of adrenal CORT, governed by nonlinear activation of the adrenal by ACTH. Levels of plasma CORT were assumed to depend upon levels of adrenal CORT and the metabolic clearance rate of plasma CORT only. The above considerations result in a system of equations2.1a
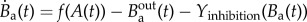
and2.1b
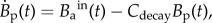
where *t* is time, *B*_a_ is the concentration of adrenal CORT, *A* is the concentration of plasma ACTH, 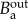 is the level of CORT diffusing out of the cell, 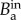 is the level of CORT diffusing into the plasma, *B*_p_ is the concentration of plasma CORT and *C*_decay_ is a parameter governing the metabolic clearance rate of plasma CORT. *f* is a function representing Michaelis–Menten activation kinetics2.2
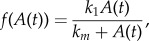
and *Y*_inhibition_ is an equation that represents the specific mechanism of inhibition of CORT synthesis for each of the four considered models

model I (no inhibition)2.3a
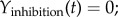


model II (inhibition)2.3b
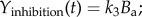


model III (delayed inhibition)2.3c
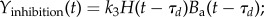


model IV (disinhibition)2.3d
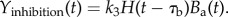
Here H is a Heaviside function assuming that the time delay (*τ*_p_) in the increase in plasma CORT (figure 2) is due to its diffusion out of the adrenal, then
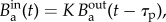
where *K* ≤ 1 [27]. From here, we may rearrange equations (2.1*a*) and (2.1*b*) to express the level of adrenal CORT (*B*_a_) in terms of the level of plasma CORT (*B*_p_)2.4
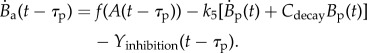


### 2.3. Assessing goodness of fit through integrating the mathematical model and experimental data

To test the validity of our four candidate models, for each model we substituted experimentally measured levels of plasma ACTH (*A*) and plasma CORT (*B*_p_) (both interpolated using piecewise cubic Hermite interpolating polynomials (pCHip)) in place of their equivalent variables in equation (2.4). Parameters in equation (2.4) were then optimized by minimizing the least-squares error (LSE) between experimentally measured and model-predicted adrenal CORT (*B*_a_):2.5
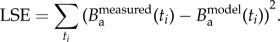


The model whose output produces the best goodness of fit in comparison with experimentally measured levels of adrenal CORT might be considered the optimal model. However, in general, the accuracy of a model (i.e. reduction in LSE score) scales with the number of free parameters. For our four candidate models, the number of free parameters increases from 3 (model I), to 4 (model II) and 5 (models III and IV). Therefore, to accurately compare models, we penalized the LSE according to the Akaike information criterion (AIC), which describes the trade-off between the goodness of fit of the model and the model complexity (i.e. number of parameters) [28]:2.6
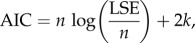
where *n* is number of data points, and *k* is number of model parameters. Because AIC effectively offers a relative estimate of the information lost, the model with the lowest score gives the best representation of the data and is thus considered most likely. We also calculated *p*-values between LSE arrays for model pairs using Wilcoxon's rank-sum method, and used the Bonferroni correction to account for multiple comparisons.

## 3. Results and discussion

If our hypothesis—that CORT levels within the adrenal are important for regulating steroidogenesis over the timescales of both the basal ultradian rhythm and the response to acute stress—is a valid one, then levels of CORT within the adrenal cortex must themselves be *dynamic* over these time frames. To determine whether this is in fact the case, we performed three different experiments: the first a 10 min acute noise stress that activates the whole HPA axis [24,25]; the second a constant CRH infusion which activates only the pituitary–adrenal subsystem to generate an ultradian rhythm in both ACTH and CORT [2]; and the third an i.v. pulse of ACTH which activates only the adrenal gland [7]. We collected the adrenal glands at regular intervals throughout each of the three experiments and measured CORT levels within the adrenal gland, in addition to plasma levels of both ACTH and CORT (figure 2).

As can be seen in figure 2*a*–*c*, each experiment resulted in a rapid pulse of ACTH and subsequent increase in plasma CORT, and this was accompanied by a pulsatile pattern of CORT within the adrenal itself. It is also apparent that the peak levels of plasma CORT response do not scale linearly with the peak levels of plasma ACTH (figure 2*d*), but are in fact reduced from the level that would be expected from a purely linear response. Given that the levels of plasma CORT observed during severe acute stressors are much greater than those observed within our experiments in this study [24,25,29,30], it is unlikely that this nonlinear reduction in CORT results from a saturation effect. Therefore, these observations suggest that, in addition to the activation of CORT synthesis by ACTH, there also exist additional mechanisms regulating the level of CORT synthesis within the adrenal itself.

To explore the potential intra-adrenal mechanisms regulating the level of CORT synthesis and secretion, we used experimentally measured levels of plasma ACTH and plasma CORT as inputs into each of the four candidate mathematical models represented schematically in figure 3, where model I represents the null hypothesis of no intra-adrenal feedback (no inhibition); model II represents non-delayed CORT negative feedback (inhibition), motivated by the presence of GR in the adrenal [13,14]; model III represents delayed CORT negative feedback (delayed inhibition), motivated by the fact that the CORT–GR interaction, and subsequent inhibition, may be dependent on intermediate steps not explicitly modelled, resulting in a time delay; and model IV represents a transient block of the intra-adrenal inhibition (disinhibition).

**Figure 4.**
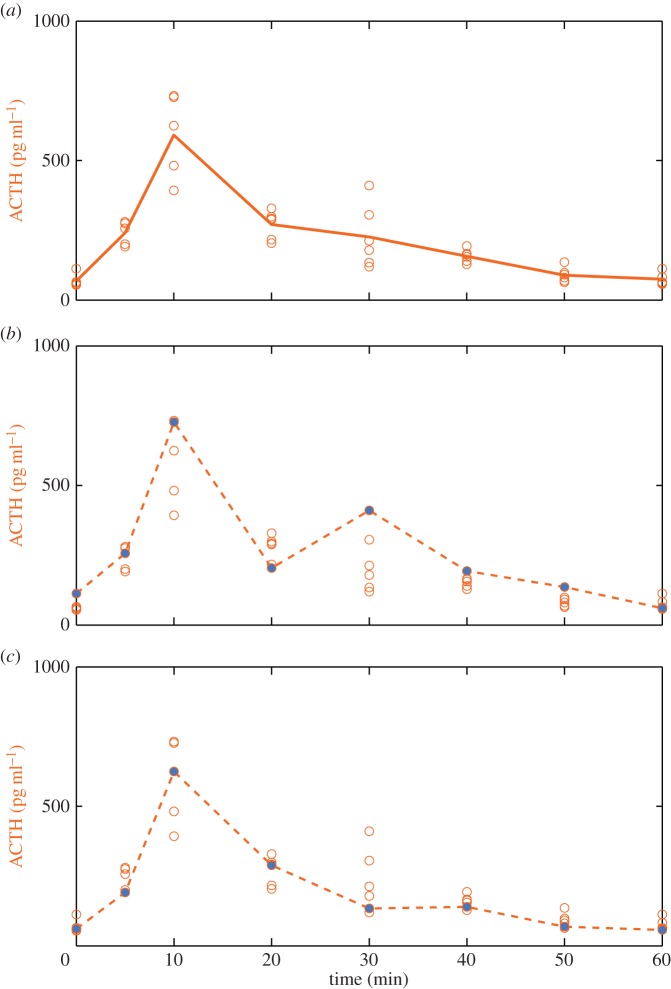
(*a*) Mean ACTH profile for the noise stress experiment and (*b*,*c*) two exemplar time-course trajectories created from a random selection of experimental data points.

**Figure 5.**
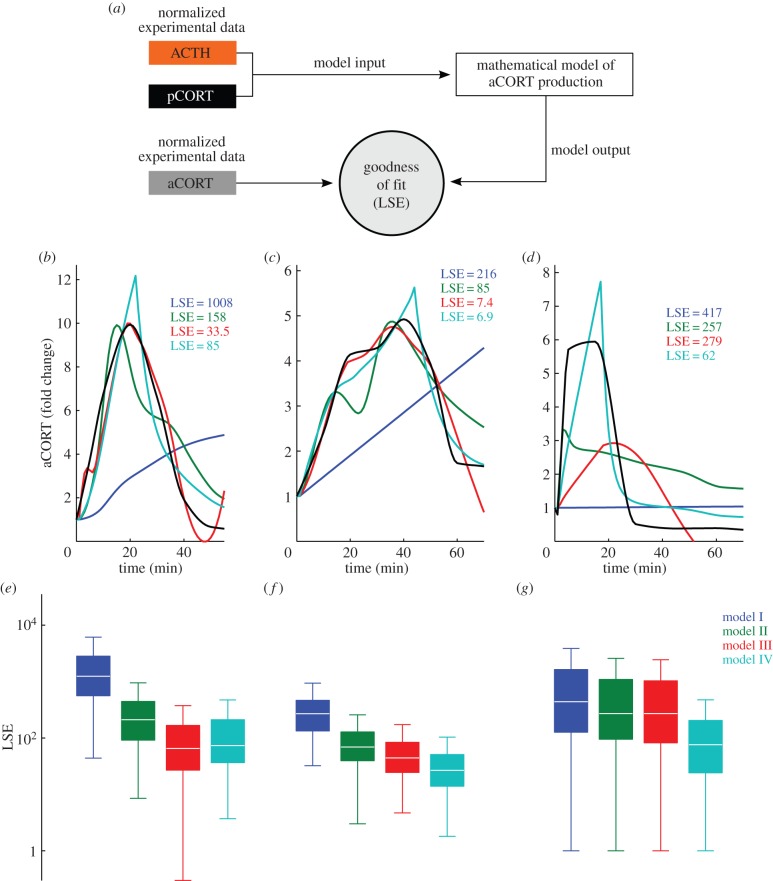
(*a*) Schematic of the fitting procedure used to determine the most likely model from our four candidate models. (*b*–*d*) Exemplar time profiles of the adrenal CORT dynamics predicted by the four candidate models for the (*b*) noise stress, (*c*) constant CRH and (*d*) ACTH pulse experiments. In each panel, the black line represents the normalized experimental adrenal CORT data, the blue line represents model I (no inhibition), the green line represents model II (inhibition), the red line represents model III (delayed inhibition) and the turquoise line represents model IV (disinhibition). Data are represented as fold induction of time 0 min. For each model fit, the least-squares error (LSE) is shown. (*e*–*g*) Box plots of LSE calculated for all 400 trajectories for each model for the (*e*) noise stress, (*f*) constant CRH and (*g*) ACTH pulse experiments. For the noise stress experiment, all pair wise comparison *p*-values are < 0.05, except for *p*_III,IV_ = 0.17. For the constant CRH experiment, all pair wise comparison *p*-values are < 0.05. For the ACTH pulse experiment, all pair wise comparison *p*-values are < 0.05, except for *p*_II,III_ = 2.4.

Because our experimental data consisted of measurements from individual animals at each time point, instead of using the average time profiles for ACTH and plasma CORT as model inputs, we created 400 time course trajectories using data points selected at random from individual experimental measurements of each hormone (figure 4). Each ACTH and plasma CORT time course trajectory was then normalized to the respective hormone level at time zero, and these values were interpolated using pCHip and used as inputs to the four mathematical models. To assess the fit of each model, we compared the experimentally measured and model-predicted adrenal CORT profiles by computing the LSE for each fit. A schematic of this process is shown in figure 5*a*. Examples of the fit between the experimentally measured and model-predicted adrenal CORT profiles are presented in figure 5*b*–*d*, along with the corresponding LSE value for each fit. Computing the fit for all 400 time course trajectories results in an LSE distribution for each model, as shown in figure 5*e*–*g*. To control for differences in model complexity, we also computed the AIC (table 1).

**Table 1.** Akaike information criterion (AIC) for models I–IV and the three experiments.

	noise stress	constant CRH	ACTH pulse
model I	178	100	134.5
model II	82	7.2	103
model III	19	−22	105
model IV	26	−57	16

We found it straightforward to reject the null hypothesis of no intra-adrenal feedback inhibition (model I), reflected in the visually poor fit to the experimental data for all three experimental paradigms and the corresponding LSE values (figure 5*b*–*g*; blue line). The inclusion of adrenal CORT-dependent inhibition (model II) resulted in a dramatic improvement in the match between the experimentally measured and model-predicted levels of adrenal CORT, reflected by the order of magnitude decrease in the LSE values for the acute noise stress and constant CRH experiments (figure 5*b,c*,*e*,*f*; green line). However, for the ACTH pulse experiment, there was a poor fit between the experimentally measured and model-predicted adrenal CORT data (figure 5*d,g*; green line). This suggests that a CORT-dependent inhibitory mechanism does not fully capture the intra-adrenal CORT dynamics when the adrenal is directly activated by ACTH.

We then extended the simple model of adrenal CORT-dependent inhibition (model II) to incorporate either a delay in the onset of adrenal CORT negative feedback (model III), or a transient disinhibition of this CORT negative feedback (model IV). For the acute noise stress experiment, the best fit occurs for model III, which has the lowest median LSE and AIC scores (figure 5*b*,*e*; red line and table 1). On the other hand, for both the constant CRH and ACTH pulse experiments, the median LSE and AIC values are lowest for model IV (figure 5*c*,*d*,*f*,*g*; turquoise line and table 1). It is important to note, however, that for the noise stress experiment, the LSE and AIC values for model IV are a close second to model III (figure 5*b*,*e*; turquoise line and table 1). This is also evident when comparing the LSE values for the two models (*p*_III,IV_ = 0.17), suggesting that, in fact, both model choices have a similar ability to explain the observed dynamics for the acute noise stress experiment.

While displaying the LSE for each model choice as a box plot (figure 5*e*–*g*) enables us to ascertain the best overall model for a given experimental paradigm, this analysis approach does not inform us about the best model choice for a specific given trajectory. To assess this, we also considered the LSE for each model on a trajectory-by-trajectory basis and then ranked each model according to its LSE value (table 2). As can be seen from table 2, the results of this trajectory-by-trajectory analysis are in agreement with our analysis of the LSE distributions in figure 5: for the noise stress study, model III has the lowest LSE (i.e. best fit) for 58% of the trajectories, compared with model IV which is the best fit for 37.5% of the trajectories; for the constant CRH and ACTH pulse experiments, model IV has the lowest LSE (i.e. best fit) for 86% and 98.75% of the trajectories, respectively.

**Table 2.** Proportion of 400 trajectories for which each model choice was optimal (based upon LSE) for each of the three experiments.

	noise stress	constant CRH	ACTH pulse
model I	0	0	0.0025
model II	0.045	0.035	0.005
model III	0.58	0.105	0.005
model IV	0.375	0.86	0.9875

**Figure 6.**
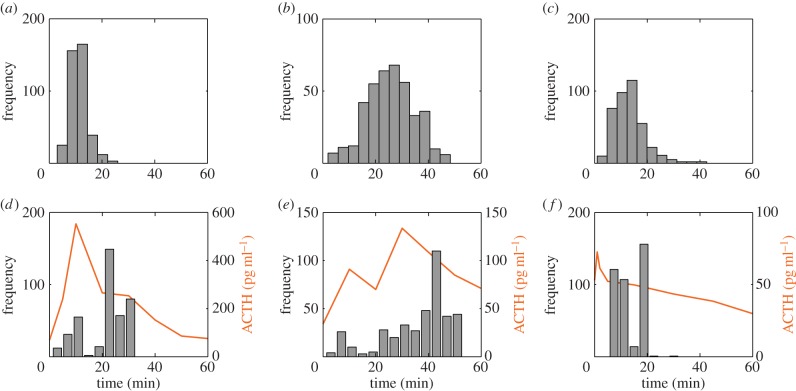
Distribution of optimal delay times for model III (delayed inhibition) for all 400 trajectories for the (*a*) noise stress, (*b*) constant CRH and (*c*) ACTH pulse experiments. Histogram of optimal duration of block of inhibition times for model IV (disinhibition) for all 400 trajectories for the (*d*) noise stress, (*e*) constant CRH and (*f*) ACTH pulse experiments. The average time profile of ACTH for all 400 trajectories (orange) is shown for the three experiments for comparison with the optimal duration of block of inhibition times.

In the case of model III (delayed inhibition), we computed the optimal delay time obtained by fitting the model to each of the 400 trajectories and plotted the distribution for each experiment (figure 6*a*–*c*). While the distribution of optimal delay times for the noise stress experiment is tightly clustered around 10–15 min (figure 6*a*), the distributions for the constant CRH and ACTH pulse experiments are more widely spread (figure 6*b*,*c*). This is consistent with our findings that model III provides a good fit for the noise stress experiment, but not for the constant CRH or ACTH pulse experiments. Interestingly, this optimal delay time for the noise stress experiment is consistent with the time taken for plasma CORT to activate GR in target tissues [31]. In addition, we have observed a rapid phosphorylation of GR (which is a marker of GR activation) within the adrenal following a rise in adrenal CORT (2014, unpublished data). This supports the hypothesis that CORT-dependent intra-adrenal inhibition occurs via a GR-dependent signalling pathway.

In the case of model IV (disinhibition), we computed the optimal duration of the block time obtained by fitting the model to each of the 400 trajectories and plotted the distribution for each experiment (figure 6*d*–*f*). In addition to this, we also plotted the average plasma ACTH for the 400 trajectories. Although the distribution of block time duration varies with each experiment, a consistent finding across all three experiments is that the peak in the distribution occurs around 15 min after the peak in plasma ACTH. This implicates a role for plasma ACTH in transiently disinhibiting (e.g. temporarily blocking) the adrenal CORT negative feedback mechanism.

Although, overall, model IV provides the optimal fit for the constant CRH and ACTH pulse experiments, in the case of the noise stress experiment, model III is the optimal fit. The reason for this difference is not clear, but it is possible that, alongside activation of the HPA axis, hypothalamic activation of the sympathetic nervous system during stress may introduce addition levels of regulation within the adrenal [32] that may, in turn, affect the dynamics of the adrenal response to ACTH. This suggests that mechanisms within the adrenal regulating steroidogenesis may change according to the nature of the ACTH stimulus (e.g. basal versus stress).

ACTH regulation of CORT synthesis involves both genomic and more rapid non-genomic mechanisms (figure 1*c*). At the genomic level, ACTH-mediated PKA activation leads to an increase in steroidogenic gene transcription and protein expression [6]. There is evidence that glucocorticoids can inhibit the transcription of steroidogenic genes via a mechanism involving GR-induced transcription of DAX-1, a repressor of StAR and MC2R transcription [33]. In addition, ACTH can antagonize glucocorticoid-induced inhibition of StAR transcription by inhibiting DAX-1 transcription [33]. This provides support for the concept of a GR-mediated intra-adrenal negative feedback mechanism, and a role for both ACTH and CORT in regulating steroidogenesis within the adrenal gland. However, given the timescales of these genomic signalling processes, it is unlikely that they underlie the rapid intra-adrenal regulation described in this study.

In addition to regulating steroidogenesis at the genomic level, GR has also been shown to associate with the catalytic subunit of PKA and regulate its activity [34]. Because the rapid non-genomic effect of ACTH on CORT synthesis involves PKA-mediated activation of proteins involved in cholesterol metabolism, a critical part of the CORT synthesis pathway, it is possible that GR-mediated regulation of PKA activity underlies the rapid intra-adrenal regulation proposed in this study.

Glucocorticoids are well known to regulate their own synthesis via rapid feedback inhibition at the level of the anterior pituitary and the brain. In this study, we have shown that an additional level of glucocorticoid autoregulation may exist within the adrenal gland itself which operates over the timescale of both the ultradian rhythm and the acute stress response. These multiple feedback mechanisms within the HPA axis have likely evolved to maintain a balance between reactivity and control. On the one hand, the body needs to respond rapidly to stress, but, on the other hand, it must avoid levels of glucocorticoid spiralling out of control, resulting in downregulation and subsequent inactivity of the system as a whole. The complexity of these networks raises the potential for their breakdown in disease [35]. Indeed, changes in the ultradian rhythm of CORT and the response to stress have been described in a number of pathological conditions [35], and abnormalities in intra-adrenal steroidogenic pathways have recently been implicated in the development of adrenal tumours [36]. Motivated by our work, further studies are necessary to elucidate the molecular components involved in regulating this intra-adrenal inhibitory mechanism.

## Data accessibility

Data used in this paper may be downloaded from the University of Bristol Research Data Repository.

## References

[1] Windle RJ Wood SA Lightman SL Ingram CD 1998 The pulsatile characteristics of hypothalamo-pituitary–adrenal activity in female Lewis and Fischer 344 rats and its relationship to differential stress responses. Endocrinology 139, 4044–4052. 9751481 10.1210/endo.139.10.6238

[2] Walker JJ 2012 The origin of glucocorticoid hormone oscillations. PLoS Biol. 10, e1001341 (10.1371/journal.pbio.1001341) 22679394 PMC3367982

[3] Kalsbeek A 2012 Circadian rhythms of the Hypothalamo-pituitary–adrenal (HPA) axis. Mol. Cell. Endocrinol. 349, 20–29. (10.1016/j.mce.2011.06.042) 21782883

[4] de Kloet ER 2004 Hormones and the stressed brain. Ann. NY Acad. Sci. 1018, 1–15. (10.1196/annals.1296.001) 15240347

[5] Arakane FJ 1997 Phosphorylation of steroidogenic acute regulatory protein (StAR) modulates its steroidogenic activity. J. Biol. Chem. 272, 32 656–23 662. (10.1074/jbc.272.51.32656) 9405483

[6] Lin D 1995 Role of steroidogenic acute regulatory protein in adrenal and gonadal steroidogenesis. Science 267, 1828–1831. (10.1126/science.7892608) 7892608

[7] Spiga F Liu Y Aguilera G Lightman SL 2011 Temporal effect of adrenocorticotrophic hormone on adrenal glucocorticoid steroidogenesis: involvement of the transducer of regulated cyclic AMP-response element-binding protein activity. J. Neuroendocrinol. 23, 136–142. (10.1111/j.1365-2826.2010.02096.x) 21083631 PMC3189260

[8] Jones MT Hillhouse EW Burden JL 1977 Structure–activity relationships of corticosteroid feedback at the hypothalamic level. J. Endocrinol. 74, 415–424. (10.1677/joe.0.0740415) 303680

[9] Dallman MF 1987 Characterization of corticosterone feedback regulation of ACTH secretion. Ann. NY Acad. Sci. 512, 402–414. (10.1111/j.1749-6632.1987.tb24976.x) 2831781

[10] Walker JJ Terry JR Lightman SL 2010 Origin of ultradian pulsatility in the hypothalamic–pituitary–adrenal axis. Proc. R. Soc. B 277, 1627–1633. (10.1098/rspb.2009.2148) PMC2871854 20129987

[11] Sarabdjitsingh RA 2010 Stress responsiveness varies over the ultradian glucocorticoid cycle in a brain-region-specific manner. Endocrinology 151, 5369–5379. (10.1210/en.2010-0832) 20861228

[12] Rankin J Walker JJ Windle R Lightman SL Terry JR 2012 Characterizing dynamic interactions between ultradian glucocorticoid rhythmicity and acute stress using the phase response curve. PLoS ONE 7, e30978 (10.1371/journal.pone.0030978) 22363526 PMC3283588

[13] Loose DS Do YS Chen TL Feldman D 1980 Demonstration of glucocorticoid receptors in the adrenal cortex: evidence for a direct dexamethasone suppressive effect on the rat adrenal gland. Endocrinology 107, 137–46. (10.1210/endo-107-1-137) 6247134

[14] Briassoulis G Damjanovic S Xekouki P Lefebvre H Stratakis CA 2011 The glucocorticoid receptor and its expression in the anterior pituitary and the adrenal cortex: a source of variation in hypothalamic–pituitary–adrenal axis function; implications for pituitary and adrenal tumors. Endocrinol. Pract. 17, 941–948. (10.4158/EP11061.RA) PMC3652405 21742609

[15] Kontula K Pomoeli UM Gunsalus GL Pelkonen R 1985 Glucocorticoid receptors and responsiveness of normal and neoplastic human adrenal cortex. J. Clin. Endocrinol. Metab. 60, 283–289. (10.1210/jcem-60-2-283) 2981241

[16] Peron FG Monclowa F Dowman RI 1960 Studies on the possible inhibitory effect of corticosterone on corticosteroidogenesis at the adrenal level in the rat. Endocrinology 67, 379–388. (10.1210/endo-67-3-379) 14431931

[17] Carsia RV Malamed S 1979 Acute self-suppression of corticosteroidogenesis in isolated adrenocortical cells. Endocrinology 105, 911–914. (10.1210/endo-105-4-911) 225159

[18] Langecker H Lurie R 1957 Inhibition of corticotropin secretion by steroids. Acta Endocrinol. 25, 54–58. 13434697

[19] Jones MT Stockham MA 1966 The effect of previous stimulation of the adrenal cortex by adrenocorticotrophin on the function of the pituitary–adrenocortical axis in response to stress. J. Physiol. 184, 741–750. 4290068 10.1113/jphysiol.1966.sp007945 PMC1357613

[20] Richards JB Pruitt RL 1957 Hydrocortisone suppression of stress-induced adrenal 17-hydroxycorticosteroid secretion in dogs. Endocrinology 60, 99–104. (10.1210/endo-60-1-99) 13384388

[21] Black WC Crampton RS Verdesca AS Nedelikovic RI Hilton JG 1961 Inhibitory effect of hydrocortisone and analogues on adrenocortical secretion in dogs. Am. J. Physiol. 201, 1057–1060. 13869668 10.1152/ajplegacy.1961.201.6.1057

[22] Stockham MA 1964 Changes of plasma and adrenal corticosterone levels in the rat after repeated stimuli. J. Physiol. 173, 149–159. 14205027 10.1113/jphysiol.1964.sp007448 PMC1368885

[23] De Souza EB van Loon GR 1982 Stress-induced inhibition of the plasma corticosterone response to a subsequent stress in rats: a nonadrenocorticotropin-mediated mechanism. Endocrinology 110, 23–33. (10.1210/endo-110-1-23) 6274619

[24] Spiga F 2009 Blockade of the V(1b) receptor reduces ACTH, but not corticosterone secretion induced by stress without effecting basal hypothalamic–pituitary–adrenal axis activity. J. Endocrinol. 200, 273–283. (10.1677/JOE-08-0421) 19008333

[25] Spiga F 2009 Effect of vasopressin 1b receptor blockade on the hypothalamic–pituitary–adrenal response of chronically stressed rats to a heterotypic stressor. J. Endocrinol. 200, 285–291. (10.1677/JOE-08-0425) 19074473

[26] Park SY 2013 Constant light disrupts the circadian rhythm of steroidogenic proteins in the rat adrenal gland. Mol. Cell. Endocrinol. 371, 114–123. (10.1016/j.mce.2012.11.010) 23178164

[27] Papaikonomou P 1977 Rat adrenocortical dynamics. J. Physiol. 265, 119–131. 191596 10.1113/jphysiol.1977.sp011708 PMC1307811

[28] Burnham KOP Anderson DR 2004 Multimodel inference: understanding AIC and BIC in model selection. Soc. Methods Res. 33, 261–304. (10.1177/0049124104268644)

[29] Chacón F 2005 24-hour changes in ACTH, corticosterone, growth hormone, and leptin levels in young male rats subjected to calorie restriction. Chronobiol. Int. 22, 253–265. (10.1081/CBI-200053522) 16021842

[30] Vahl TP 2005 Comparative analysis of ACTH and corticosterone sampling methods in rats. Am. J. Physiol. Endocrinol. Metab. 289, E823–E828. (10.1152/ajpendo.00122.2005) 15956051

[31] Conway-Campbell BL 2007 Proteasome-dependent down-regulation of activated nuclear hippocampal glucocorticoid receptors determines dynamic responses to corticosterone. Endocrinology 148, 5470–5477. (10.1210/en.2007-0585) 17690167

[32] Jasper MS Engeland WC 1997 Splanchnicotomy increases adrenal sensitivity to ACTH in nonstressed rats. Am. J. Physiol. 273, E363–E368. 9277390 10.1152/ajpendo.1997.273.2.E363

[33] Gummow BM Scheys JO Cancelli VR Hammer GD 2006 Reciprocal regulation of a glucocorticoid receptor-steroidogenic factor-1 transcription complex on the Dax-1 promoter by glucocorticoids and adrenocorticotropic hormone in the adrenal cortex. Mol. Endocrinol. 20, 2711–2723. (10.1210/me.2005-0461) 16857744

[34] Doucas V 2000 Cytoplasmic catalytic subunit of protein kinase A mediates cross-repression by NF-kappa B and the glucocorticoid receptor. Proc. Natl. Acad. Sci. USA 97, 11 893–11 898. (10.1073/pnas.220413297) PMC17265 11027313

[35] Lightman SL Terry JR 2014 The importance of dynamic signalling for endocrine regulation and drug development: relevance for glucocorticoid hormones. Lancet Diabet. Endocrinol. 2, 593–599. (10.1016/S2213-8587(13)70182-7) 24731665

[36] Cao Y 2014 Activating hotspot L205R mutation in PRKACA and adrenal Cushing's syndrome. Science 344, 913–917. (10.1126/science.1249480) 24700472

